# Metabolic Modulation of Macrophage Function Post Myocardial Infarction

**DOI:** 10.3389/fphys.2020.00674

**Published:** 2020-06-30

**Authors:** Mingyue Zhao, Dennis Ding-Hwa Wang, Xiaojing Liu, Rong Tian

**Affiliations:** ^1^Laboratory of Cardiovascular Diseases, Regenerative Medicine Research Center, West China Hospital, Sichuan University, Chengdu, China; ^2^Mitochondria and Metabolism Center, Department of Anesthesiology and Pain Medicine, University of Washington, Seattle, WA, United States; ^3^Division of Cardiology, Department of Medicine, University of Washington, Seattle, WA, United States; ^4^Department of Cardiology, West China Hospital, Sichuan University, Chengdu, China

**Keywords:** myocardial infarction, mitochondrial function, immunometabolism, macrophage, monocyte

## Abstract

Macrophages are key components of innate immunity, and they play critical roles in heart health and diseases. Following acute myocardial infarction (MI), infiltrating macrophages undergo drastic phenotypic transition from pro-inflammatory in the early stage to pro-healing in the late stage. Transcriptome analyses of macrophage in the infarct zone show a time-dependent reprogramming of mitochondrial and metabolic functions, which parallels the changes of macrophage function. These observations suggest that mitochondrial and metabolic targets could be exploited for therapeutic opportunities. In this article, we reviewed the recent work on immunometabolic features of macrophage over the MI time continuum. In addition, we summarized currently proposed mitochondrial pathways involved in the functional polarization of macrophage and discussed their potential relevance to the outcome of MI. We expect that these findings will stimulate further investigations in metabolic modulation of innate immunity in the post-MI setting, which could ultimately lead to new strategies for therapy.

## Introduction

Ischemic heart disease is a leading cause of morbidity and mortality in developed countries. Cell death caused by myocardial infarction (MI) or ischemic/reperfusion (IR) injury invokes the recruitment of immune cells to the infarcted myocardium, which triggers inflammatory response in the early stage followed by wound healing and eventual scar formation (Van der Borght and Lambrecht, 2018; Bajpai et al., 2019). It has been shown that excessive inflammation and/or inadequate wound healing leads to poor clinical outcome after MI (Jia et al., 2019). Innate immune cells, particularly macrophages and monocytes, play a primary role in the tissue damage/repair process. Emerging studies suggest that macrophage function is intricately linked to its metabolic profile (Palmieri et al., 2017; Yurdagul et al., 2020). After acute MI (AMI), the majority of resident macrophages in the infarct zone die and are subsequently replenished by macrophages derived from circulating monocytes (Heidt et al., 2014; Ma et al., 2018). These macrophages undergo complex phenotypic changes that are central to post-MI healing. For example, infiltration and activation of macrophages in the infarcted region lead to strong inflammatory response, cytokine release, and further cell death immediately after MI (Mezzaroma et al., 2011; Chen et al., 2019). As the tissue repair commences, macrophage population evolves from pro-inflammatory to pro-healing, which promotes collagen deposition and extracellular matrix remodeling and scar formation (DeLeon-Pennell et al., 2017; Honold and Nahrendorf, 2018). Moreover, recent studies suggest that macrophages are critical mediators of the functional benefit associated with adjunctive cell therapy post-MI (de Couto et al., 2015; Vagnozzi et al., 2020). Therefore, regulatory mechanisms governing the transitions of macrophage functions during the post-MI period has recently become a fervid area of investigation. In this mini review, we will summarize the recent advances regarding the role of mitochondrial metabolism in regulating the phenotypic changes of macrophage post-MI.

## Metabolic Reprogramming of Macrophage Post-MI

**FIGURE 1:**
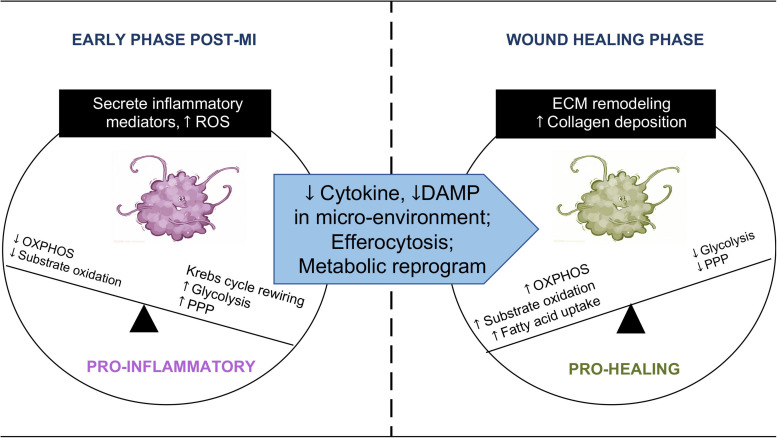
Immunometabolic transition of macrophage post-MI. Macrophages undergo an immunometabolic transition post-MI. At the early phase, macrophages secrete pro-inflammatory cytokines in the injured myocardium. Their energy metabolism shifts toward glycolysis and increased flux through the pentose phosphate pathway (PPP), accompanied by TCA cycle rewiring and a high level of mtROS. Substrate oxidation and ATP synthesis through OXPHOS are reduced. When the infarcted region enters the wound healing phase, macrophages become phagocytotic. Changes in the microenvironment, efferocytosis, and metabolic reprogramming promote the phenotype transition to pro-healing. Macrophages at this stage revert to oxidative metabolism and engage in collagen deposition and remodeling of the extracellular matrix (ECM).

A global rewiring of metabolic pathways takes place in macrophages during the transition from a quiescent state to an activated state (Kelly and O’Neill, 2015). A hallmark of pro-inflammatory macrophage is a metabolic shift from oxidative metabolism toward glycolysis to meet the bioenergetic and biosynthetic demand (Rodriguez-Prados et al., 2010; Figure 1). In the case of pathogen invasion, upregulation of glycolysis rapidly generates ATP for activated macrophages. Furthermore, increased glycolytic intermediates feed into the pentose phosphate pathway (PPP) to produce NADPH, which is used to generate reactive oxygen species (ROS), through NADPH oxidase reaction, for bactericidal function (West et al., 2011a). On the other hand, mitochondrial oxidative metabolism in pro-inflammatory macrophages is suppressed, and the tricaboxylic acid (TCA) cycle flux is disrupted and rewired (Figure 1; Jha et al., 2015; Lampropoulou et al., 2016). In a sterile condition, infiltrating monocyte/macrophage in the infarct zone post-MI mounts a similar inflammatory response in the early stage post-MI (Lee et al., 2012). For instance, damage-associated molecular patterns (DAMPs) released by the dead cells activate toll-like receptors (TLRs) and promote glycolytic polarization of macrophages within the injured heart (Williams et al., 2018). The metabolic profile of macrophages in the infarct zone is not well described. However, a recent study examining macrophage transcriptome in the post-MI hearts showed a robust reprogramming of mitochondrial genes during the transition from tissue injury to repair (Mouton et al., 2018), suggesting that mitochondrial function could also be central to macrophage phenotype and cardiac remodeling post-MI.

Following MI, clearance of apoptotic cells by immune cells, i.e., efferocytosis, is a critical step leading to inflammation resolution and tissue repair (Serhan and Savill, 2005; Wan et al., 2013; Gordon, 2016). Distinct from microbial phagocytosis, efferocytosis after MI has been shown to fill the phagocytic macrophage with a heavy substrate load almost equal to the phagocyte itself (Zhang et al., 2019). The presence of metabolic cargo is accompanied by a marked elevation of oxygen consumption in phagocytic macrophages relative to non-efferocytes. In parallel, fatty acid oxidation (FAO) and oxidative phosphorylation (OXPHOS) are upregulated in phagocytic macrophages, while glycolysis decreases as cytokines and DAMPs decline in the microenvironment. These changes constitute a reversal of metabolic profile as the inflammation resolves (Park et al., 2011; Figure 1). The importance of mitochondria in macrophage is supported by a recent study showing poor wound healing and increased cardiac rupture in mice with myeloid-specific deficiency of Complex III in the electron transport chain (ETC) (Zhang et al., 2018). While these findings introduce a new role of mitochondria in cardiac repair and remodeling, they raise many questions in regard to mechanisms and therapeutic implications. In the section below, we will discuss briefly the proposed mechanisms linking mitochondrial metabolism to macrophage phenotype.

## Role of Mitochondrial Metabolism in Modulating Macrophage Function

**FIGURE 2:**
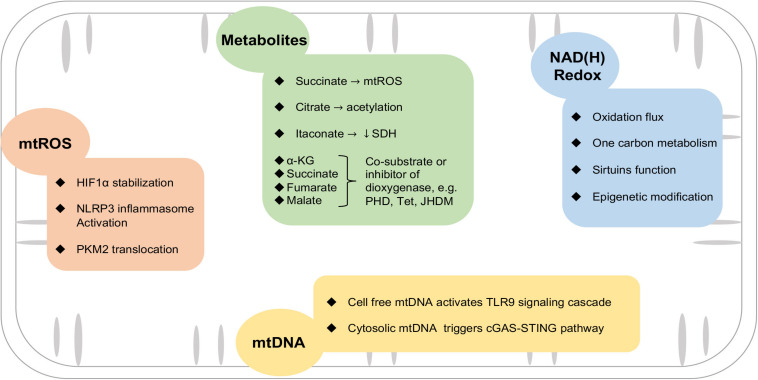
Mitochondrial mechanisms involved in macrophage activation. Mitochondria provide triggers and mediators of the inflammatory response in macrophages via mtROS, metabolites, NAD(H) redox balance, and leakage of mtDNA. See text for details. HIF1α, hypoxia-inducible factor 1α; SDH, succinate dehydrogenase; NLRP3, nod-like receptor family pyrin domain containing 3; cGAS-STING, GMP-AMP synthase/stimulator of interferon genes; PKM2, pyruvate kinase M2; JHDM, Jumonji domain-containing histone demethylase; PHD, prolyl hydroxylases; Tet, T5-methylcytosine hydroxylases.

Besides producing ATP for the cell, mitochondria play multiple regulatory roles in cellular signaling, redox balance, cell growth, and survival (Zhou and Tian, 2018; Ritterhoff et al., 2020). Changes in mitochondrial function have been observed in activated macrophages, but the molecular mechanisms connecting mitochondrial function and macrophage phenotypes are not fully understood. In this section, we will summarize emerging findings on the role of mitochondrial ROS, intermediary metabolism, and NAD(H) redox state in modulating macrophage function, as well as the role of mitochondrial DNA (mtDNA) in triggering inflammatory response (Figure 2).

### Mitochondrial ROS

Reactive oxygen species is a by-product of mitochondrial respiratory activity. Under physiologic conditions, electron leakage in Complexes I and III of the ETC is a major source of ROS in mitochondria (mtROS) (Wong et al., 2017). Enzymes localized on the outer membrane of mitochondria, such as monoamine oxidase A, also generate ROS in activated macrophages, which has been thoroughly reviewed (Cathcart and Bhattacharjee, 2014). In pro-inflammatory macrophages, parallel studies show that impairment of TCA flux results in succinate accumulation via succinate oxidation by succinate dehydrogenase (SDH). Under conditions of reduced OXPHOS, succinate oxidation generates high mitochondrial membrane potential and drives the reverse electron transport (RET) at Complex I to produce ROS (Mills et al., 2016; Robb et al., 2018). In macrophages stimulated by lipopolysaccharide (LPS), succinate oxidation boosts mtROS generation and enhances interleukin-1β (IL-1β) production (Mills et al., 2016). It has been reported that the NOTCH signaling pathway is required to reprogram mitochondrial metabolism, thus leading to mtROS generation and pro-inflammatory gene expression (Xu et al., 2015). Blocking Complex I by alternative oxidase (AOX) or rotenone or scavenging mtROS by Mito-TEMPO inhibits inflammatory phenotype in macrophages (Jin et al., 2014; Scialo et al., 2017). Interestingly, blocking mtROS also reduces the population of anti-inflammatory macrophages with isoprenaline treatment (Shan et al., 2017), suggesting mtROS may modulate macrophage phenotype transition in a context-dependent fashion.

How does mtROS modulate inflammation? Increased mtROS is associated with increased gene expressions of pro-inflammatory cytokines, such as tumor necrosis factor α (TNFα) and IL-1β (van de Veerdonk et al., 2010; O’Neill and Pearce, 2016). mtROS could stabilize hypoxia-inducible factor 1α (HIF1α) through inhibiting the activity of prolyl hydroxylases (PHDs). Enhanced HIF1α is considered central to the upregulation of glycolysis and inflammatory cytokine expression (Calvani et al., 2012; Kelly and O’Neill, 2015). In monocytes and macrophages derived from atherosclerotic coronary artery disease patients, mtROS promotes dimerization and nuclear translocation of the glycolytic enzyme pyruvate kinase M2 (PKM2), phosphorylating the transcription factor signal transducer and activator of transcription (STAT3), thus boosting IL-6 and IL-1β production (Shirai et al., 2016). mtROS also serves as a key regulator of nod-like receptor family pyrin domain containing 3 (NLRP3) inflammasome activation (Afonina et al., 2017; Chen et al., 2017). In the early phase after AMI, NLRP3 senses danger signals both extracellularly and intracellularly, such as DAMPs, and assembles an inflammasome to mediate the downstream response (Mauro et al., 2019). On the other hand, mitochondrial antiviral protein (MAVS) facilitates the recruitment of NLRP3 to the mitochondria and hence enhances its oligomerization and activation by bringing it close to mtROS (Park et al., 2013; Figure 2).

### Intermediary Metabolism

Disruption of the TCA cycle in the pro-inflammatory macrophages resulted in accumulations of metabolic intermediates such as citrate, succinate, fumarate, and malate (Ryan and O’Neill, 2017). As alluded to earlier, accumulation of succinate promotes mtROS generation via SDH. Succinate has also been shown to stabilize HIF1α by inhibiting the activity of PHD, an α-ketoglutarate (α-KG)-dependent dioxygenase. α-KG, on the contrary, depletes HIF1α by promoting the activity of PHD (Tannahill et al., 2013). The activity of other dioxygenases, e.g., Jumonji domain-containing histone demethylase (JHDM) or T5-methylcytosine hydroxylases (Tet), also requires α-KG as a co-substrate and can be inhibited by succinate, fumarate, or malate (Xu et al., 2011; Sinton et al., 2019; Figure 2). A recent study showed that increased glutaminolysis drove fumarate production via the TCA cycle, leading to enhanced TNFα and IL-6 expressions via boosting H3K4 trimethylation (H3K4me3) at the respective promoter regions (Arts et al., 2016). Moreover, α-KG derived from glutaminolysis is shown to be important for the alternative activation of macrophage via epigenetic regulations of pro-reparative genes (Liu et al., 2017).

Citrate transported from mitochondria can be converted to acetyl-CoA by ATP-citrate lyase (ACLY) in the cytosol. This mechanism has been implicated in the regulation of histone acetylation of cancer cells (Wellen et al., 2009). While upregulation of ACLY is observed in pro-inflammatory macrophages, it remains to be determined whether increased histone acetylation is responsible for the associated increase of inflammatory mediators, e.g., nitric oxide (NO), ROS, and prostaglandin E2 (PGE2) (Infantino et al., 2013). Another metabolite derived from the TCA cycle is itaconate, which is converted from aconitate (O’Neill and Artyomov, 2019). A recent study suggests that itaconate functions as an anti-inflammatory mediator in metabolic remodeling of macrophages via inhibition of SDH during IR injury (Lampropoulou et al., 2016).

In contrast to the pro-inflammatory macrophages, oxidative metabolism is robust in alternatively activated macrophages which phenotypically resemble reparative macrophages. During the clearance of injured tissues, phagocytes gain lipid load via efferocytosis, which likely stimulates fatty acid utilization. A recent study suggests that fatty acid is the preferred substrate in pro-healing macrophages after cardiac injury (Zhang et al., 2019). Increased fatty acid metabolism and associated activation of peroxisome proliferator-activated receptor (PPAR α/β/δ) in macrophages appear to play an important role in polarizing macrophages into a pro-reparative phenotype (Chawla, 2010). This led to the conjecture that the lipid load macrophages acquired via efferocytosis may potentially stimulate fatty acid utilization. Interestingly, elevated utilization of glucose is also necessary for pro-reparative macrophage polarization, and this may be mediated through increased production of UDP-GlcNAc (Jha et al., 2015; Huang et al., 2016). A major limitation in interpreting these observations is that most studies are done *in vitro*. Technical innovations, such as isolation procedures that preserve *in vivo* phenotype or genetic strategies for specific lineage tracing or perturbations, are required to allow for metabolic analysis of macrophages *in vivo*.

### NAD(H) Redox Balance

Nicotinamide adenine dinucleotide (NAD^+^) accepts electrons from glycolysis, TCA cycle, and β-oxidation to form NADH, which then feeds its reducing potential to the ETC for OXPHOS. Therefore, NAD(H) redox is a major determinant of mitochondrial function and *vice versa*. Decreased OXPHOS lowers the NAD^+^/NADH ratio and reduces the availability of NAD^+^ for other NAD^+^-dependent proteins, such as poly ADP-ribose polymerase (PARP), cyclic ADP-ribose synthase, and sirtuins (Verdin, 2015; Katsyuba and Auwerx, 2017; Figure 2). Recently, Zhang et al. demonstrated that mitochondrial Complex III deficiency in macrophages impaired transcriptional activation of IL-10 induced by efferocytosis post-MI, resulting in poor healing and rupture of the infarcted ventricle. The defect was attributed to impaired Sirt1 function and could be rescued by supplementing nicotinamide mononucleotide (NMN), a NAD^+^ precursor (Zhang et al., 2019), suggesting that NAD(H) redox imbalance caused by mitochondrial dysfunction is an important regulatory mechanism of immune response. Impairment of the Sirt2 function due to NAD^+^ depletion leads to the hyperacetylation of α-tubulin, which in turn promotes colocalization of apoptosis-associated speck-like protein containing a caspase recruitment domain (ASC) and NLRP3, and drives IL-1β production in macrophages (Zhang et al., 2018). NAD(H) redox balance is also a powerful regulator of epigenetics, likely via supporting the activity of sirtuin deacetylases (Class III HADCs) on histone (Halili et al., 2010; Ciarlo and Roger, 2016). Recent studies show that DNA and histone methylation also regulate macrophage function (Yu et al., 2019; Li et al., 2020). One carbon metabolism, which supplies the methyl group for methylation, is regulated by mitochondrial function and the NAD^+^/NADH ratio (Tibbetts and Appling, 2010; Kuhl et al., 2017). Together, these observations substantiate the role of NAD(H) redox in the modulation of innate immunity (Tibbetts and Appling, 2010; Zhang et al., 2019).

### mtDNA Is a Potential Pro-inflammatory Mediator of Post-MI Macrophage

Increased levels of circulating cell-free mtDNA (CCF-DNA) have been observed in a number of disease conditions including MI (West and Shadel, 2017; Nakayama and Otsu, 2018). Due to the structural features resembling prokaryotic DNA, mtDNA is a potent pro-inflammatory trigger for immune cells in sterile inflammation (Collins et al., 2004) and is widely accepted as an integral member of the DAMPs. Hypomethylated CpG-rich mtDNA serves as an endogenous ligand for TLR9 to trigger the innate immune system governed by the NLRP3 inflammasome in response to cellular stress, infection, and AMI injury (West et al., 2011b). In the canonical pathway of sterile inflammation, upon sensing extracellular mtDNA released during necrotic tissue death, TLR9 recruits adaptor proteins MyD88 and TRIF to activate transcription factors NF-kB and NLRP3 to promote production, maturation, and secretion of pro-inflammatory cytokines, such as IL-1β, IL-6, and IL-18, to amplify the inflammatory signals (Chen and Nunez, 2010; Nakayama and Otsu, 2018).

The presence of mtDNA in the cytosol also triggers inflammation. Defective autophagy in bone-marrow-derived macrophages (BMDMs) resulted in enhanced cytosolic translocation of mtDNA via the mitochondrial membrane permeability transition pore (MPTP) in an mtROS- and NLRP3-dependent fashion to activate caspase 1, promoting IL-1β and IL-18 secretions (Nakahira et al., 2011). Key players in autophagy, Beclin-1 and LC3, have been found critical in the regulation of macrophage phagocytosis and efferocytosis, as well as the clearance of apoptotic cells, inflammatory resolution, and tissue repair following MI (Konishi et al., 2012; Heckmann et al., 2017; Heckmann and Green, 2019). More recently, cytidine/uridine monophosphate kinase 2 (CMPK2)-dependent mtDNA synthesis was shown to be required for NLRP3 inflammasome activation in LPS-primed macrophages, in that the newly synthesized mtDNA was oxidized by mtROS and translocated to the cytosol to activate NLRP3 inflammasome via direct binding (Zhong et al., 2018). Together, these studies demonstrate the co-dependence of mtDNA’s cytosolic translocation and the activation of NLRP3/caspase I axis of macrophage in a pro-inflammatory milieu such as the early phase of the post-MI myocardium.

GMP-AMP synthase (cGAS)/stimulator of interferon genes (STING) is another cytosolic mtDNA-sensing pathway which governs the gene expressions of interferons, such as CD14, CXCL10, and IRFs. In dendritic cells and macrophages, cGAS can be activated by cytoplasmic mtDNA to resist infection (Carroll et al., 2016; Chen et al., 2016). In the context of mtDNA stress induced by transcription factor A, mitochondrial (TFAM) deficiency in BMDMs, aberrant mtDNA packaging results in the cytosolic translocation of mtDNA. Upon sensing cytosolic fragmented mtDNA, cGAS activates STING, which then activates TBK1, resulting in the translation of interferon genes (West et al., 2015). Recently, cGAS-dependent mtDNA sensing has been demonstrated to mediate macrophage polarization. cGAS^–/–^ mice displayed an improved outcome after MI, which is associated with an augmented reparative macrophage population. However, a loss of cGAS does not alter the mRNA levels of canonical cytokines (Cao et al., 2018). Together, these findings suggest that cGAS/STING is a pro-inflammatory pathway parallel to the TLR9/NF-kB axis to sense aberrant cytosolic mtDNA in post-MI macrophages.

## Conclusion and Perspectives

As discussed above, mitochondrial metabolism is a key regulator of macrophage response. Substrate metabolism, mtROS, and NAD(H) redox state influence macrophage phenotype through epigenetic, transcriptional, and posttranscriptional mechanisms. Microenvironment, such as oxygen and nutrient availability, DAMPs, and metabolic cargo from cell debris, also modulates functional properties of macrophage via mitochondria and metabolic mechanisms. These observations not only provide mechanistic links between mitochondrial responses and the functional transition of macrophages during the remodeling after MI but also suggest a novel class of targets for therapy. Given the broad implication of innate immunity in human diseases, these findings will drive future work to identify molecular mechanisms connecting mitochondria and immune cell functions in a wide variety of diseases.

## Author Contributions

MZ wrote the manuscript. DW and RT edited the manuscript. RT and XL supervised the work and reviewed the manuscript. All authors contributed to the article and approved the submitted version.

## Conflict of Interest

The authors declare that the research was conducted in the absence of any commercial or financial relationships that could be construed as a potential conflict of interest.
